# Cardiovascular MRI: A valuable tool to detect cardiac source of emboli in cryptogenic ischemic strokes

**DOI:** 10.1002/brb3.1620

**Published:** 2020-04-18

**Authors:** Yousef Mohammad, Tariq Alhoqbani, Rashed Alfaqih, Lamees Altamimi, Abdullah Alotaibi, Abdulelah AlMousa, Fayez El Shaer, Fawaz Al‐Hussain

**Affiliations:** ^1^ Department of Internal Medicine College of Medicine King Saud University Riyadh Saudi Arabia; ^2^ Department of Cardiology College of Medicine King Saud University Riyadh Saudi Arabia; ^3^ College of Medicine King Saud University Riyadh Saudi Arabia

**Keywords:** cardiac‐emboli, cardiovascular MRI, stroke, transesophageal echocardiogram

## Abstract

**Objectives:**

Despite a thorough work‐up including transesophageal echocardiography, 20%–30% of stroke etiology remains cryptogenic. Transesophageal echocardiogram is considered the gold standard procedure to detect cardiac or aortic sources of emboli. In the recent years, cardiovascular MRI has emerged as a noninvasive, sound, and reliable modality to image morphological and functional abnormalities. In this study, we compared none contrast cardiovascular MRI to transesophageal echocardiogram, in the ability to detect cardiovascular source of embolus in cryptogenic ischemic strokes.

**Methods:**

A series of 24 patients who were labeled, after a thorough stroke work‐up, as having cryptogenic stroke, were examined with both transesophageal echocardiogram and noncontrast cardiovascular MRI to assess for cardiac or aortic source of emboli. The cardiologist who interpreted the transesophageal echocardiograms was blinded to the results of cardiovascular MRI. At the same time, the radiologist who interpreted the cardiovascular MRI was also blinded to the results of transesophageal echocardiogram. The cardiac lesions, with potential source of emboli that were assessed in our study included left ventricular thrombus, atrial septal aneurysm, and aortic atherosclerotic disease. The ability of cardiovascular MRI to identify potential source of cardiac embolus was then compared to that of transesophageal echocardiogram.

**Results:**

Transesophageal echocardiogram detected ascending or arch aortic atherosclerotic plaque in 14 of the 24 patients. Other abnormalities detected include two atrial septal aneurysms and two left ventricular thrombus. Cardiovascular MRI was able to identify aortic atheroma in 13 patients; as well as three atrial septal aneurysms and two left ventricular thrombus. The accuracy of cardiovascular MRI to detect aortic atheroma, atrial septal aneurysm or left ventricular thrombus was great; 96%, 95.83%, and 100%, respectively.

**Conclusion:**

This small study suggests that, in patients with cryptogenic stroke, cardiovascular MRI is comparable to transesophageal echocardiogram in detecting cardiac and aortic source of emboli.

## Graphical abstract

Etiology of stroke remains unknown in 20%–30% of patients. Transesophageal echocardiogram has been considered the gold standard procedure to detect cardiac sources of emboli. Cardiovascular MRI has emerged as a valuable tool to detect cardiac source of emboli in cryptogenic ischemic strokes.

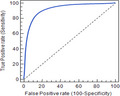

## 1 INTRODUCTION

Stroke is a leading global health concern. It remains the leading cause of long‐term disability in adults and drags behind only cardiovascular diseases in causing death worldwide; accounting for 5% of all disability (GBD 2016 DALYs and HALE Collaborators, 2017) and 10% of all deaths (GBD 2016 Causes of Death Collaborators, 2017). In fact, 25% of people over the age of 25 years will develop stroke in their life time (The GBD 2016 Lifetime Risk of Stroke Collaborators, 2018). Moreover, the global burden of stroke is expected to increase (GBD 2013 Writing Group and GBD 2013 Stroke Panel Experts Group, 2015).

Hence stroke prevention, both primary and secondary, remains of utmost importance; but effective secondary stroke prevention depends on the stroke mechanism. Systematic approach to identify etiology of stroke has been proposed, and many procedures are considered standard of care. For instance, thrombophilia screen will assess for thrombogenic condition; carotid Doppler and CT/MR angiogram of neck will check for extracranial vessel disease; CT/MR angiogram of brain will screen for intracranial large vessel disease; and 24‐hr holter monitor and transthoracic echocardiogram (TTE) will uncover potential cardiac source of stroke. It has been ascertained that 20%–25% of ischemic strokes are caused by cardiac emboli (Kelley and Minagar, 2003; Díaz, 2012). Cardio‐embolic strokes have been also associated with worse outcome (Marini et al., 2005). Moreover, ascending and aortic arch plaques have been established as an important cause of embolic ischemic strokes. It has also been verified that transesophageal echocardiogram (TEE), instead of TTE, is the gold standard and the most sensitive tool to identify cardiac and aortic source of emboli (Pearson, Labovitz, Tatineni, & Gomez, 1991). Nevertheless, despite a thorough work‐up, including TEE, 30% of stroke etiology remains cryptogenic (Yaghi, Kamel, & Elkind, 2017). Cardiovascular MRI (CMRI) has emerged as a highly accurate modality to image morphological and functional abnormalities. However, data on the merit of CMRI to detect cardiac or aortic source of emboli remain limited. In this study, we sought to compare noncontrast CMRI with TEE, in the capability of detecting cardiovascular source of embolism in cryptogenic ischemic strokes.

## 2 MATERIAL AND METHODS

### 2.1 Subjects

The study was conducted at King Khalid university hospital in Riyadh, Saudi Arabia. All patients, who were consecutively admitted with acute ischemic stroke, were evaluated by a neurologist with a formal training in cerebrovascular diseases. A thorough stroke work‐up was performed in all patients admitted to the hospital with acute ischemic stroke. The work‐up included thrombophelia panel (if patient is young), TTE, 24‐hr holter monitor, CT or MR angiogram of neck, and CT or MR angiogram of brain. The TOAST criteria were used for the classification of stroke mechanism (Adams et al., 1993); and cryptogenic stroke was defined as an ischemic stroke for which the routine work‐up could not determine the stroke mechanism. TEE was reserved to the patients whose stroke remains cryptogenic despite the above stroke work‐up and cardiac embolic source was highly suspected. All patients who were scheduled for TEE were also arranged to have a noncontrast CMRI. Both the TEE and CMRI were arranged and performed within 1 week from the stroke onset. The yield of uncovering potential cardiac source of emboli was then compared between the two modalities. The study protocol was approved by the institutional review board of the King Khalid University Hospital, and the patient's consent for CMRI was obtained before proceeding with the procedure.

### 2.2 CMRI protocol

CMRI of 1.5 Tesla units was utilized in our study (GE Signa Discovery 450 HD; GE Healthcare). The device was furnished with complete cardiac hardware and software with ECG cardiac gating utilizing multi‐channel cardiac phased‐array coils. The procedure included cine sequences (GE, FIESTA) for cardiac chambers and valvular assessment; as well as dark blood images for aortic wall examination. In this study, only the following potential cardiac source of emboli was assessed by the CMRI modality: the presence of left ventricular thrombus (LVT), the presence of atrial septal aneurysm (ASA), and the presence and degree of aortic atherosclerotic disease. The average duration of the procedure was 45 min; and tolerance was not assessed in this study since it was already verified in previous published reports. No fee for the CMRI was incurred by the patient because health care is for free in the Kingdom of Saudi Arabia.

### 2.3 TEE protocol

The equipment utilized to do the TEE was EPIQ (Philips healthcare). The imaging procedure included multiplane examination of numerous cardiac structures and pathology. Features of the TEE probe include: 2 to 7 MHz operating range, 2D matrix array with 2,500 elements, 2D live xPlane, 3D live color Doppler, and 3D xPlane. Images were obtained digitally during the test and filed for later review utilizing Xcelera digital workstation (Philips, BG). For the sake of our study, we only collected data on the presence of LVT, ASA, and aortic disease.

### 2.4 Data collection and analysis

The CMRI was interpreted by a cardiologist with formal fellowship training in the interpretation of CMRI. The cardiologist was blinded to the findings of the corresponding TEE. The TEE was assessed by a cardiologist with fellowship training in echocardiography; who was also blinded to the results of the corresponding CMRI. The presence of ascending or aortic arch atherosclerotic plaques was classified as mild, if the thickness of the plaque was <2 mm; moderate, if the thickness was 2–4 mm; or severe, if the thickness was >4 mm.

## 3 RESULTS

Between March 2018 and June 2019, a series of 24 patients, with cryptogenic stroke, were examined with both TEE and noncontrast CMRI to assess for cardiac or aortic source of embolic stroke. The mean age was 62 years; and 54.1% were males (Table 1). As anticipated, half of the study population were hypertensive or diabetic; 50% and 45.8%, respectively. Interestingly, the study population was in general heavy with a mean BMI of 26.7. Only two patients refused to undergo CMRI. Also, CMRI was contraindicated in one patient because of an implanted pacemaker.

**TABLE 1:** Demographics and clinical characteristics

Characteristics	Total population (*n* = 24) *N* (%)
Age (years)	
Mean ± *SD*	62 ± 11.1
Gender	
Male	13 (54.1)
Females	11 (45.9)
Hypertension	
Yes	12 (50)
No	12 (50)
Diabetes mellitus	
Yes	11 (45.8)
No	13 (54.2)
Coronary artery disease	
Yes	4 (16.6)
No	20 (83.4)
Smoking	
Yes	5 (20.8)
No	19 (79.2)
Dyslipidemia	
Yes	9 (37.5)
No	15 (62.5)
Body mass index	
Mean ± *SD*	26.7 ± 7.2

**FIGURE 1:**
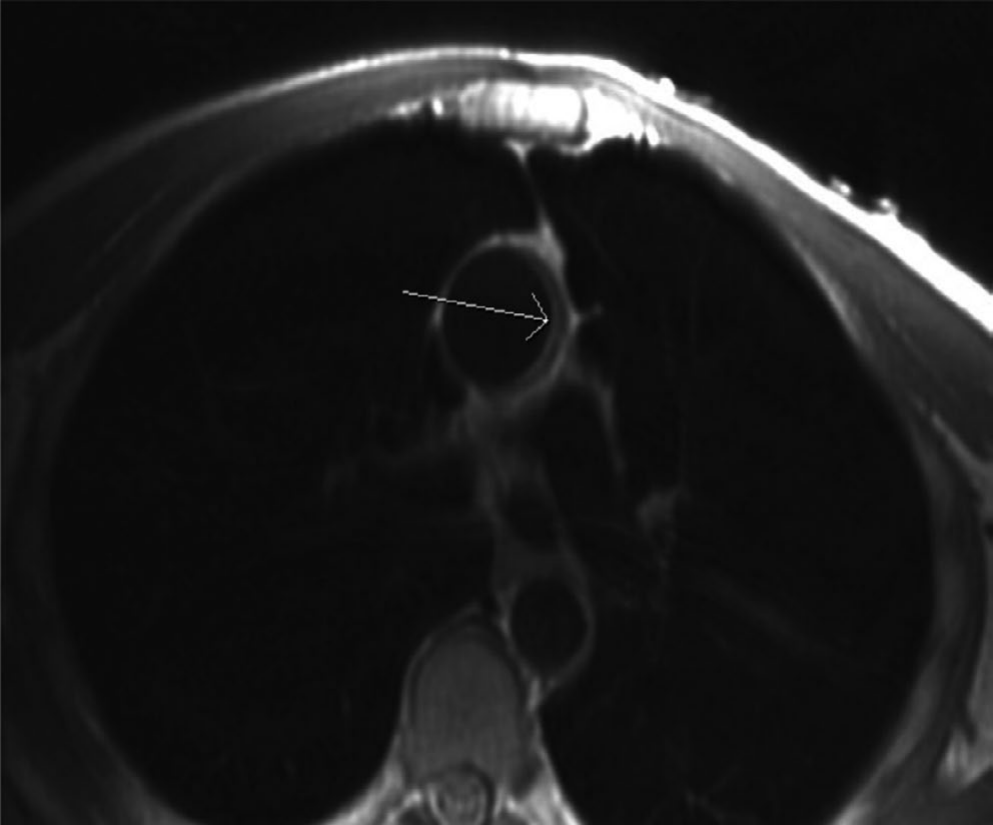
Cardiovascular MRI demonstrates significant atherosclerosis in ascending aorta (Patient # 2)

**FIGURE 2:**
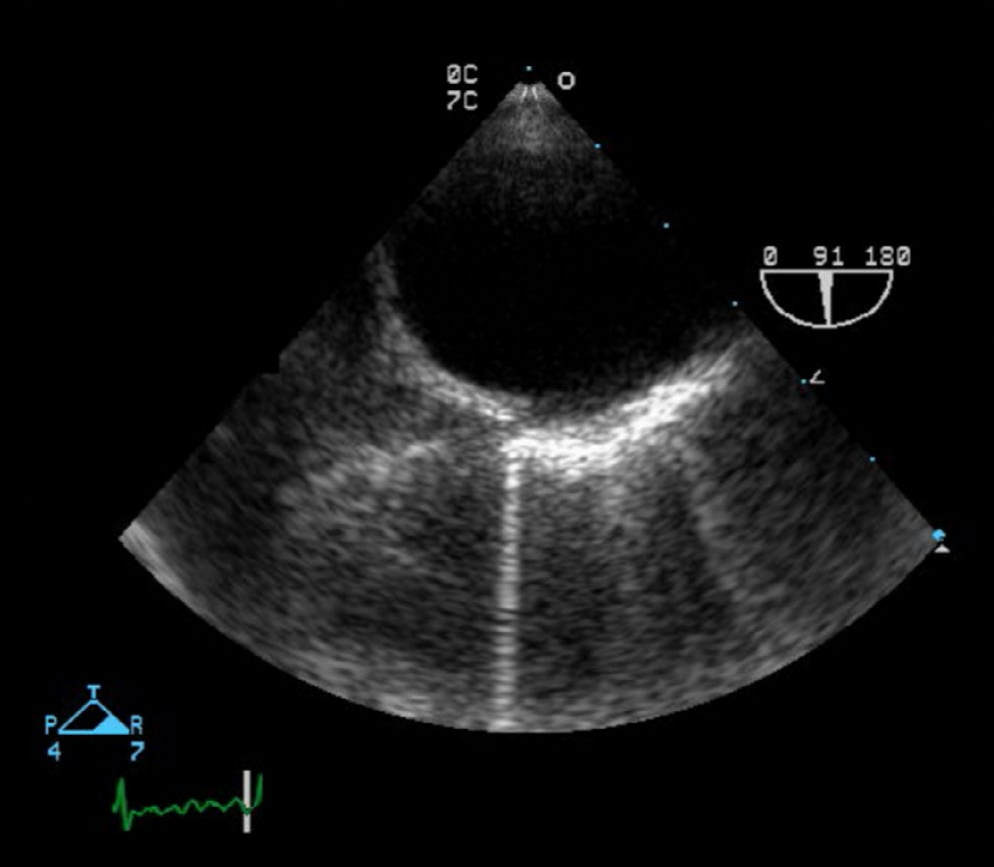
Transesophageal echocardiogram showed only minimal aortic arch atherosclerosis (Patient # 2)

TEE detected ascending or arch aortic atheroma in 14 of the 24 patients. Other abnormalities detected included 2 ASA and 2 LVT. CMRI was comparable to TEE in identifying aortic atheroma and LVT; 13 and 2, respectively (Table 2). However, it was superior to TEE in revealing ASA; 3 compared to 2. CMRI could not detect one aortic atheroma which was actually graded as only minimal by the TEE. However, there is a significant discrepancy between the two modalities in the estimation of the degree of aortic atheroma; four of the aortic atheroma that were graded as mild by TEE, were rated as moderate or severe by the CMRI (Figures 1 and 2).

**TABLE 2:** Frequency of aortic atheroma, LVT, and ASA in the CMRI and TEE

Patient	CMRI			TEE		
	Aortic atheroma	ASA	LVT	Aortic Atheroma	ASA	LVT
1	+	**−**	**−**	+	**−**	**−**
2	+++	**−**	**−**	+	**−**	**−**
3	+++	**−**	**−**	+	**−**	**−**
4	−	**−**	**−**	−	**−**	**−**
5	++	**−**	**+**	+	**−**	**+**
6	+	**+**	**−**	+	**+**	**−**
7	−	**−**	**−**	−	**−**	**−**
8	−	**−**	**−**	−	**−**	**−**
9	+	**−**	**−**	+	**−**	**−**
10	−	**−**	**−**	−	**−**	**−**
11	−	**−**	**−**	−	**−**	**−**
12	−	**−**	**−**	−	**−**	**−**
13	++	**−**	**−**	++	**−**	**−**
14	−	**−**	**−**	−	**−**	**−**
15	++	**−**	**−**	+	**−**	**−**
16	+++	**−**	**−**	+++	**−**	**−**
17	+	**+**	**−**	+	**+**	**−**
18	+	**−**	**−**	+	**−**	**−**
19	−	**−**	**+**	−	**−**	**+**
20	+	**−**	**−**	+	**−**	**−**
21	−	**−**	**−**	++	**−**	**−**
22	−	**−**	**−**	−	**−**	**−**
23	−	**−**	**−**	−	**−**	**−**
24	+	**+**	**−**	++	**−**	**−**

Note

Mild aortic atheroma = +; moderate aortic atheroma = ++; severe aortic atheroma = +++.

Abbreviations: −, Absent; +, Present; ASA, atrial septal aneurysm; CMRI, cardiovascular MRI; LVT, left ventricular thrombus; TEE, transesophageal echocardiogram.

**FIGURE 3:**
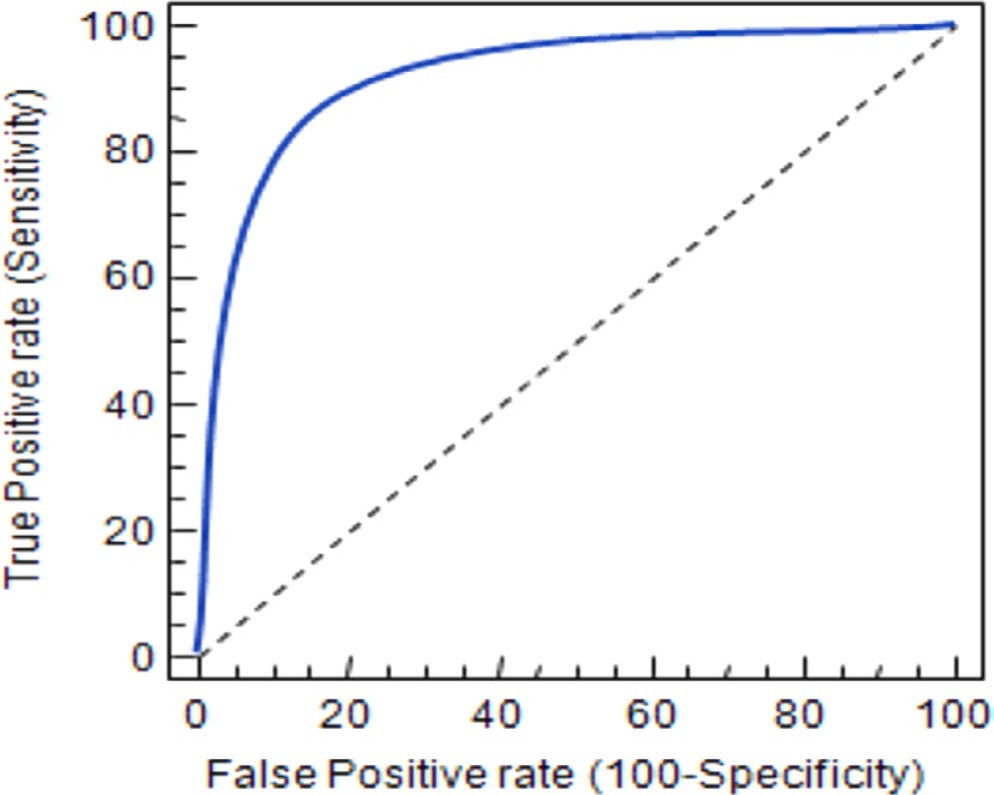
ROC curve as examined by the cardiovascular MRI

As illustrated by the sensitivity and specificity values, our results showed a high degree of correlation of the CMRI, when compared to TEE, for the detection of potential cardio‐embolic sources in cryptogenic ischemic strokes. The sensitivity of CMRI in the diagnosis of Aortic atheroma, Atrial Septal Aneurysm, and Left ventricular thrombus was 92.86%, 100%, and 100%, respectively; and the specificity was 100%, 95.45%, and 100%, respectively (Tables 3 and 4). Moreover, the ROC curve showed a high predictive accuracy of the CMRI; with an AUC of 0.773, 95% CI = 51.6–76.8 (Figure 3).

**TABLE 3:** Summary of the correlations between CMRI and TEE findings

Diagnosis	CMRI	TEE		*p* value
		Correlates	Does not correlate	
Presence of AA	13	13	0	<.0001
Absence of AA	11	10	1	.0069
Total	24	23	1	<.0001
Presence of ASA	3	2	1	<.0001
Absence of ASA	21	21	0	.0074
Total	24	23	1	.0286
Presence of LVT	2	2	0	.0074
Absence of LVT	24	22	0	.0209
Total	24	24	0	.0013

Note

*p* < .05 is statistically significant.

Abbreviations: AA, aortic atheroma; ASA, atrial septal aneurysm; CMRI, cardiovascular MRI; LVT, left ventricular thrombus; TEE, transesophageal echocardiogram.

**TABLE 4:** Accuracy of the CMRI for the detection of AA, ASA, and LVT, with TEE as reference

Diagnostic test evaluation	AA		ASA		LVT	
	Value	95% CI	Value	95% CI	Value	95% CI
Sensitivity	92.86%	66.13% to 99.82%	100.00%	15.81% to 100.00%	100.00%	15.81% to 100.00%
Specificity	100.00%	71.51% to100.00%	95.45%	77.16% to 99.88%	100.00%	84.56% to 100.00%
Positive predictive value	100.00%	0.00	66.67%	22.76% to 93.14%	100.00%	0.00
Negative predictive value	91.67%	62.46% to 98.64%	100.00%	0.00	100.00%	0.00
Accuracy	96.00%	79.65% to 99.90%	95.83%	78.88% to 99.89%	100.00%	85.75% to 100.00%

Abbreviations: 95% CI = 95% confidence interval; AA, aortic atheroma; ASA, atrial septal aneurysm; CMRI, Cardiovascular MRI; LVT, left ventricular thrombus; TEE, transesophageal echocardiogram.

## 4 DISCUSSION

Both ASA and LVT are major cardiac source of ischemic stroke (Celaj & Prabhakaran, 2019); and our study showed that CMRI was equivalent to TEE in the ability to identify these two cardiac lesions. This is in agreement with the published report by Liberman et al. who showed the sensitivity of CMRI is equivalent to TEE in the ability to detect ASA and intracardiac thrombus (Liberman et al., 2017). In fact, in the study published by Scrichai et al. CMRI was found superior to both TTE and TEE in revealing LVT (Srichai et al., 2006). Atherosclerotic plaque in the ascending aorta and aortic arch is another cardiovascular source of ischemic stroke (French Study of Aortic Plaques in Stroke Group et al., 1996). CMRI was able to identify four moderate to severe aortic diseases that were estimated as only mild by TEE. Hence, our study indicates that CMRI might be more sensitive to TEE in assessing for atherosclerotic plaque in the aorta. Our results are consistent with earlier reports that examined the value of CMRI in detecting aortic source of emboli. For example, both Zahuranec et al. and Fayad et al. showed that CMRI compares well with TEE in examining for aortic athrosclerosis (Fayad, Nahar, Fallon, Goldman, & Aguinaldo, 2000; Zahuranec et al., 2012). The value and superiority of CMRI over that of TTE, in cryptogenic ischemic strokes, has already been established by Baher et al. (2014). In his study, which included 106 patients, the rate of cryptogenic stroke was reduced by 39% when CMRI was added to the TTE. In our study, however, we compared the yield of CMRI, in cryptogenic ischemic strokes, to that of TEE; and our results, which were in accord with the report by Liberman et al., supported the CMRI merit in this stroke subtype.

PFO, LV ejection fraction, and other cardiac lesions with potentials for cardio‐embolic stroke were not assessed in our research; which is a major limitation of our study. The sample size was small (only 24 subjects); which is another limitation of the study.

TEE has been considered the gold standard technique to evaluate for cardiac source of emboli. The proximity of the TEE probe to the aorta and left atrium makes it highly accurate in assessing for PFO, ASA, aortic atherosclerosis, and left atrial appendage (Isbell & Dent, 2004; Silverman & Manning, 1998). When compared to TTE, TEE has been shown, by multiple prior studies, to be much more superior in detecting all potential cardiac source of stroke (Cujec, Polasek, Voll, & Shuaib, 1991; Pop et al., 1990; Zenker et al., 1988). However, it is an unpleasant procedure that is associated with throat pain and annoying taste from the topical oropharyngeal anesthetic. It is also relatively more invasive procedure that requires conscious sedation and is associated with multiple complications such as arrhythmias, mucosal injury, and esophageal perforation (Min et al., 2005). Additionally, it is contraindicated in medically unstable patients and in patients with esophageal stricture or severe cervical degenerative joint disease. Thus, there has been serious interest for another device that would provide similar valuable information as does the TEE, but is less invasive.

In recent years, CMRI has emerged as a promising tool in this field. In 1982, the first three‐dimensional MR image of the heart was introduced; and since its introduction, the technique has advanced extensively. CMRI has been illustrated to not only be a noninvasive procedure but also be excellent device to evaluate the cardiac morphology, ventricular function, myocardial perfusion, valvular pathology, and coronary artery disease (Bruder et al., 2009, 2013; Karamitsos, Francis, Myerson, Selvanayagam, & Neubauer, 2009). Its use, however, is contraindicated in patients with non‐MRI compatible implanted devices, pacemakers, defibrillators, metallic devices (Hsu, Parker, & Puranik, 2012), patients who are too heavy or large to fit in the machine and those with severe claustrophobia. Other limiting elements include physical discomfort from lengthy procedure, annoying noise, and cost. At any rate, CMRI has emerged as a potential alternative to TEE in patients who could not tolerate TEE or in patients whom their TEE could not uncover cardiovascular source of emboli.

Since its introduction to the medical field, the handful of studies that compared its value to TEE in evaluating for cardiac source of ischemic stroke, has been encouraging. In turn, the results of our small pilot study are consistent with the results of the reported studies and ascertain the merit of CMRI in cryptogenic strokes. This is especially true when examining for aortic atherosclerosis or intracardiac mass, such as LVT. The utilization of CMRI in ischemic stroke will result in further reduction in cryptogenic stroke proportion; consequently, this will greatly optimize stroke prevention measures.

In conclusion, CMRI is a promising vital tool in exposing cardiac source of emboli in cryptogenic strokes. Hence, we believe that a larger study to intensively examine the potential clinical application of CMRI in cryptogenic stroke is strongly compelling.

## CONFLICT OF INTEREST

The authors declare they have no conflict of interest.

## AUTHOR'S CONTRIBUTIONS

Yousef Mohammad contributed to the study design and concept, analysis of the data, and drafting/revising the manuscript. Tariq Alhogbani contributed to the analysis of the results, interpretation of the data, and drafting/revising the manuscript. Rashed Alfagih contributed to the analysis of the results, interpretation of the data, and drafting/revising the manuscript. Abdullah Alotaibi contributed to the data collection, study concept, and drafting/revising the manuscript. Abdulelah AlMousa contributed to the data collection, study design, and drafting/revising the manuscript. Lamees Altamimi contributed to the data collection, study concept, results analysis and drafting/revising the manuscript. Fayez El Shaer contributed to the analysis of the results, interpretation of the data, and drafting/revising the manuscript. Fawaz Al‐Hussain contributed to the study design, analysis of the data, and drafting/revising the manuscript.

## Data Availability

The data used to support the findings of this study is available from the corresponding author upon request.
